# Dentists for the Army and Navy

**Published:** 1881-12

**Authors:** 


### ARTICLE V.

### *Dentists for Army and Navy.*

About the year 1850 the subject of appointing dentists in the Army and Navy was first brought forward in this country. About this date the question was also agitated in France and England, but in America only the matter has been given the prominence and character to which those interested claim it is entitled. It was not until 1861 that the American Dental Convention appointed a committee to urge the Government to take the matter under consideration. In 1868 a bill calling for the appointment of dentists in both branches of the national service was laid before Congress by Senator Hamlin, of Maine. This was, in reality, the first effort of consequence made; but despite a strong exertion on the part of the advocates of the reform to get the bill through, it was referred to the committee on Military and Naval Affairs, from whom it received only passing notice. During the Forty-second Congress a second bill was presented by the Hon. DeWitt Townsend, advocatnig dental appointments to the Military and Naval Academies, but, as in the previous instance, it was passed over to the committee, who gave it little or no consideration. A dentist has been appointed to the Naval Academy at Annapolis, with the rank of Assistant Surgeon, and this is really the only movement the Government has made favorable to appointments of the character referred to. President Lincoln favored the movement, and many dentists of standing and eminent physicians considered it worthy of their support. The most zealous workers in the cause are, perhaps, Drs. Edward Maynard, of Washington; J. D. White, of Philadelphia; J. H. McKellops, of St. Louis; W. H. Atkinson, A. P. Merrill, and George H. Perine, of New York. The latter, in a conversation with the writer upon the subject, said: "Dental chairs have been established in most of the State medical Colleges, and the acknowledgement of dentistry as a specialty of medicine is general. Dentistry is a science, not a mere mechanical trade, and men of ability and experience have devoted themselves to the production of text-books, without which no library would be complete. Much of the opposition we have had to contend with in our efforts to have dentistry accepted as one of the medical requirements of Army and Navy practice, has had its origin in the fear that its introduction would necessitate the displacement of present incumbents or the increase of expense through the establishment of a dental bureau. A bureau would be unnecessary, nor would there be any increase of expense to the Government for the reason that it is not proposed to appoint men for the practice of dentistry only, but to select, when vacancies occur or additional appointments are found necessary, candidates from the ranks of those who have graduated from colleges where dental surgery constitutes a part of the regular course."

Lieut. H. Whiting, of the United States Marine Corps, expressed himself as follows regarding the subject: "I consider the appointment of surgeons who possess a thorough knowledge of dentistry a necessity, and I believe the Government should and will before long take decided steps toward that end. Nowhere is the want of dental treatment experienced more than on board training ships and seagoing vessels—particularly at foreign stations—and in most cases the only remedy resorted to for an aching tooth is the forceps; hence many valuable teeth are sacrificed in the absence of proper dental treatment and other and more serious disorders often follow. From men now in my command who have served in the Army I have learned that the same state of affairs exists at the military posts in the far West. I am fully convinced that much suffering is experienced by soldiers and sailors from the lack of proper treatment of diseases of the teeth. Sound teeth constitutes one of the physical requirements of men entering the service, and it is but right that the Government should bestow upon its servants the care necessary to protect their health. Neither soldiers nor sailors can afford from their limited remuneration, to pay for dental treatment, and it is unjust that a man entering the service with sound teeth should lose them for want of proper care during the term of his enlistment."

Lieut. Commander Heverman, executive officer of the receiving ship Colorado, said to the writer: "I am decidedly in favor of the appointment of surgeons in the Navy who possess a knowledge of dentistry. The care of the teeth of sailors is a matter worthy of consideration, and I am greatly in favor of the movement."

Post Surgeon Major Janeway, at present stationed at Governor's Island, stated that he had experienced the necessity of dental services in the Army, and that he had endeavored to supply the deficiency at all times so far as was in his power.

Dr. Frank H. Hamilton was one of the signers of the second petition presented to Congress appealing to that body for the enactment of a bill authorizing Army and Navy dental appointments, and he expressed himself in favor of appointments of the character above alluded to.

In 1858, the chief of the medical staff of the French Army, Baron Larrey, issued an order calling the attention of officers to the condition of the teeth of soldiers, and suggesting that something should be done to preserve them from decay and loss; and about the same period Gen. Sir De Lacy Evans endeavored to interest the British Government in the same subject, and at his instigation a circular was distributed among regimental Surgeons reminding them of the value of soldiers' teeth. As they were not supplied with the requisite instruments, and in most cases had little or no knowledge of dentistry, the effort was attended with but slight beneficial results.

At the International Medical Congress, held in London last August, Thomas Gaddes, L. D. S., of Edinburgh, read a paper on "Dental Surgery in the Army," in which he referred to the union of dentistry with medicine, and endeavored to impress his auditors with the importance of the establishment of dental surgery as one of the requirements of the Army medical department. It is understood that the same subject is to be brought before the American Medical Association at its next annual session in May, and an effort will be made to induce the authorities at Washington to give the subject consideration.

